# Puerperal Hæmorrhage Arrested by Iodine Injections

**Published:** 1869-08-15

**Authors:** 


### FOREIGN ITEMS.

***Puerperal Hæmorrhage arrested by Iodine Injections.***

M. Dupierris, of Havana, reports to the *Gazette des Hopitaux*, Paris, his experience in the use of iodine injections for the arrest of uterine hæmorrhages. Commencing as far back as 1835, with the application of the tincture of iodine upon pledgets of lint to the cervix of a cancerous uterus, and succeeding thus in arresting an otherwise uncontrollable hæmorrhage; he contined to use it to control metrorrhagia, diluting it with two volumes of water; and still later as an intra-uterine injection. He has subsequenty used the tincture of iodine frequently as an intra-uterine injection in cases of puerperal hæmorrhage, with great advantage and without accident.—*Gazette des Hopitaux.*

